# All-Solid-State Potentiometric Ion-Sensors Based on Tailored Imprinted Polymers for Pholcodine Determination

**DOI:** 10.3390/polym13081192

**Published:** 2021-04-07

**Authors:** Hisham S. M. Abd-Rabboh, Abd El-Galil E. Amr, Abdulrahman A. Almehizia, Ayman H. Kamel

**Affiliations:** 1 Chemistry Department, Faculty of Science, King Khalid University, Abha 61413, Saudi Arabia; habdrabboh@kku.edu.sa; 2 Department of Chemistry, Faculty of Science, Ain Shams University, Cairo 11566, Egypt; 3 Pharmaceutical Chemistry Department, Drug Exploration & Development Chair (DEDC), College of Pharmacy, King Saud University, Riyadh 11451, Saudi Arabia; mehizia@ksu.edu.sa; 4 National Research Center, Applied Organic Chemistry Department, Dokki, Giza 12622, Egypt

**Keywords:** all-solid-state, ion-selective electrodes (ISEs), multiwalled carbon nanotubes (MWCNTs), pholcodine, pharmaceutical analysis

## Abstract

In recent times, the application of the use of ion-selective electrodes has expanded in the field of pharmaceutical analyses due to their distinction from other sensors in their high selectivity and low cost of measurement, in addition to their high measurement sensitivity. Cost-effective, reliable, and robust all-solid-state potentiometric selective electrodes were designed, characterized, and successfully used for pholcodine determination. The design of the sensor device was based on the use of a screen-printed electrode modified with multiwalled carbon nanotubes (MWCNTs) as a solid-contact transducer. Tailored pholcodine (PHO) molecularly imprinted polymers (MIPs) were prepared, characterized, and used as sensory receptors in the presented potentiometric sensing devices. The sensors exhibited a sensitivity of 31.6 ± 0.5 mV/decade (*n* = 5, *R*^2^ = 0.9980) over the linear range of 5.5 × 10^−6^ M with a detection limit of 2.5 × 10^−7^ M. Real serum samples in addition to pharmaceutical formulations containing PHO were analyzed, and the results were compared with those obtained by the conventional standard liquid chromatographic approach. The presented analytical device showed an outstanding efficiency for fast, direct, and low-cost assessment of pholcodine levels in different matrices.

## 1. Introduction

Pholcodine (3-(2-morpholinoethyl)morphine), a derivative of morphine, was originally prepared in France in the 1950s. This modification of its chemical structure changed its pharmacological effects, as it does not cause respiratory depression, pain relief, or central nervous system (CNS) stimulation. It is also devoid of euphoric properties and the risk of addiction. It is used as an antitussive and is classified as an opioid cough suppressant [1]. Pholcodine has unproductive cough suppressing activity and is used as a mild sedative with little or no analgesic effects [2]. It has not been prescribed in the United States (US) yet, as it is classified as a Schedule I drug. In the United Kingdom (UK), it was classified as a Class B drug and officially taken off the shelves in 2008. In Canada, it has not been approved for use. In Egypt, pholcodine is classified as a substance subject to drug restrictions according to Table 3 attached to the Egyptian Drug Law No. 182 of 1960. Pholcodine can share immunoglobulin E (IgE)-binding epitope, which contains the quaternary ammonium ion (QAI) or its tertiary variety [3]. It has been shown to stimulate the production of IgE antibodies to QAI sensitization properties and thus is an alternative source for neuromuscular blocking agent (NMBA) sensitization. Different methodologies for pholcodine quantification have been reported, including nonaqueous titration [4], chemiluminescence spectrometry [5,6], absorption spectrophotometry [7], gas chromatography (GC) [8,9,10,11], high-performance liquid chromatography (HPLC) [12,13,14,15,16,17], thin-layer chromatography [18], capillary electrophoresis (CE) [19], electrochemistry [20], and ^1^HNMR-pH titration [21]. The use of electrochemical sensors, especially potential measuring sensors, has expanded greatly and now covers a wide application range in various fields [22,23,24,25,26,27,28,29,30]. 

Screen-printing technology is an advanced method of manufacturing cost-effective, compact, and portable monitoring analytical devices for on-site analysis [31,32,33]. All-solid-state potentiometric-sensing electrodes have been extensively used in the determination of different ionic species because of their high reliability, good durability, and simple operation [34,35,36,37,38]. Recently, screen-printed sensors with conductive substrates printed on the screen have received great attention. To increase the potential stability and reliability of these types of sensors, different materials with excellent electrochemical properties have been used as ion-to-electron transducers [39,40,41]. 

Molecularly imprinted polymers (MIPs) are a class of polymers that are characterized by having specific recognition sites with memory of shape, size, and function of the templates used in their preparation. MIPs have many promising advantages, including being highly selective, robust and thermally stable, inexpensive, and easy to prepare [42]. They have been widely applied in multiple fields such as stimulation [43,44,45], purification and separation [46,47], chemical and biological sensitization [48], concentration determination [49,50], and helical recognition [51]. All these advantages enable these receptors to be utilized as promising ionophores in designing ion-selective electrodes (ISEs).

The objective of this study was to develop and present cost-effective, compact, and portable monitoring potentiometric devices for analysis of pholcodine in its dosage forms and in plasma. All-solid-state screen-printed potentiometric electrodes were designed, characterized, and proposed. The potentiometric analytical device integrates the indicator polymeric membrane pholcodine ion-selective electrode with a Ag/AgCl reference electrode and a polyvinyl butyral (PVB) reference membrane. All potentiometric performances of the fabricated screen-printed sensor were investigated and evaluated.

## 2. Materials and Methods

### 2.1. Instrumentation

The potential measurements were carried out using a PXSJ-216 INESA mV/pH meter (Scientific Instrument Co., Ltd., Shangahi, China). Screen-printed platforms with carbon screen (SPCEs) modified with multiwalled carbon nanotubes (MWCNTs) (Ref. 110CNT) were purchased from DropSens (LLanera, Asturias, Spain). The platforms were made from ceramic (L34 × W10 × H0.5 mm) and silver as an electrical contact. Chronopotentiometric measurements were carried out using Autolab 204 potentiostat/galvanostat (Metrohom, Herisau, Switzerland). Pt wire was used as an auxiliary electrode. 

### 2.2. Reagents and Chemicals

Tetrahydrofuran (THF), 2-nitrophenyl octyl ether (*o*-NPOE), methacrylic acid (MAA), 2-morpholin-4-ium-4-ylethanesulfonate (MES), ethylene glycol dimethacrylate (EGDMA), benzoyl peroxide (BPO), poly(vinyl chloride) (PVC), and sodium tetrakis(3,5-bis(trifluoromethyl)phenyl) borate (NaTFPB) were obtained from Sigma-Aldrich (St. Louis, MO, USA). Ag/AgCl ink (E2414) was purchased from Ercon (Wareham, MA). Pholcodine (PHO) was kindly supplied by Amoun Pharmaceutical Co. (El-Obour City, Cairo, Egypt) with a purity >99.0%. Deionized water (18.2 MΩ cm specific resistance) from a Millipore Milli-Q system was used to prepare all solutions. 

A stock PHO solution (10^−2^ M) was prepared after dissolving 0.398 g of pure PHO solid with 0.1 M HCl in a 100 mL measuring flask. The solution was then completed to the mark with 50 mM pH 5 MES buffer. All working standard solutions (10^−8^–10^−3^ M) were prepared from the stock solution daily and prior to the measurements by appropriate dilution with MES, pH 5.

### 2.3. Sensor Construction

One hundred milligrams of the components forming the sensing membrane (8.0 wt% MIP, 2.0 wt% NaTFPB, 31.2 wt% PVC, and 58.8 wt% *o*-NPOE) was dissolved in 1.5 mL of tetrahydrofuran (THF). For reference membrane formation, the cocktail was made by dissolving 70 mg of NaCl and 78.1 mg of polyvinyl butyral (PVB) in 1 mL of methanol [52]. The indicator electrode (SPE/MWCNTs/PHO-ISE) and a solid-state Ag/AgCl reference electrode were included and integrated into the screen-printed potentiometric sensor. Fifteen microliters of the indicator membrane solution was drop-casted on the conducting carbon screen to prepare the indicator electrode. The solid-state Ag/AgCl reference electrode was prepared after adding 20 μL of the reference membrane solution on the Ag/AgCl ink electrode surface. The coated-wire indicator electrode (SPE/PHO-ISE) was prepared by the same procedure as that used for the SPE/MWCNTs/PHO-ISE but without modification with MWCNTs. Conditioning of the presented electrodes was carried out after soaking them in 10^−3^ M PHO for 4 h before measurements.

## 3. Results and Discussion

### 3.1. MIP Synthesis and Characterization

**Figure 1:**
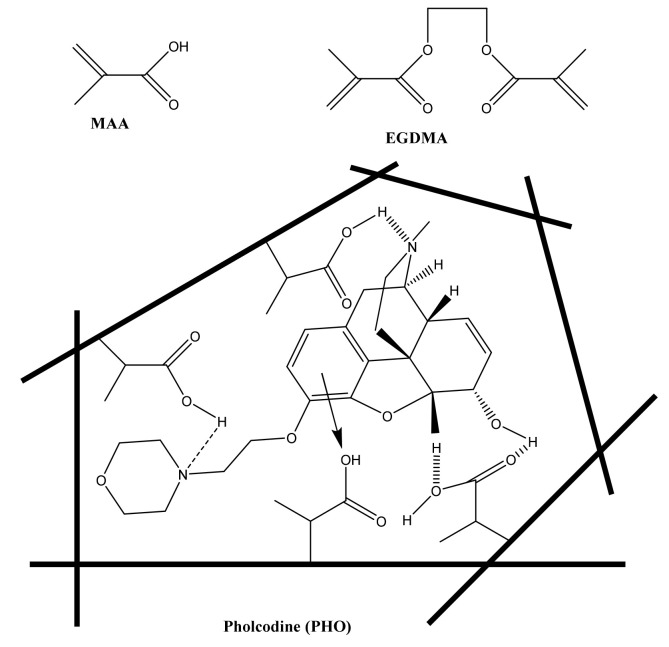
A schematic representation for the imprinting process of pholcodine.

The PHO-imprinted beads were prepared using the classical precipitation polymerization method [53]. Methacrylic acid (MAA) and ethylene glycol dimethacrylate (EGDMA) were used as a functional monomer and cross-linker, respectively. Acetonitrile was used as a porogenic solvent. In the imprinting process, the carboxyl group (COOH) in MAA interacts with the hydroxyl group and with either the tertiary amine or oxygen in the oxazine group of PHO. In addition, π–π interaction can occur between the aromatic moiety of the template with either the carboxylic group of MAA or the carbonyl group present in EGDMA. All of these types of interactions improve the binding affinity and specificity of the MIP [54]. A schematic representation of the imprinting process is shown in Figure 1. 

**Figure 2:**
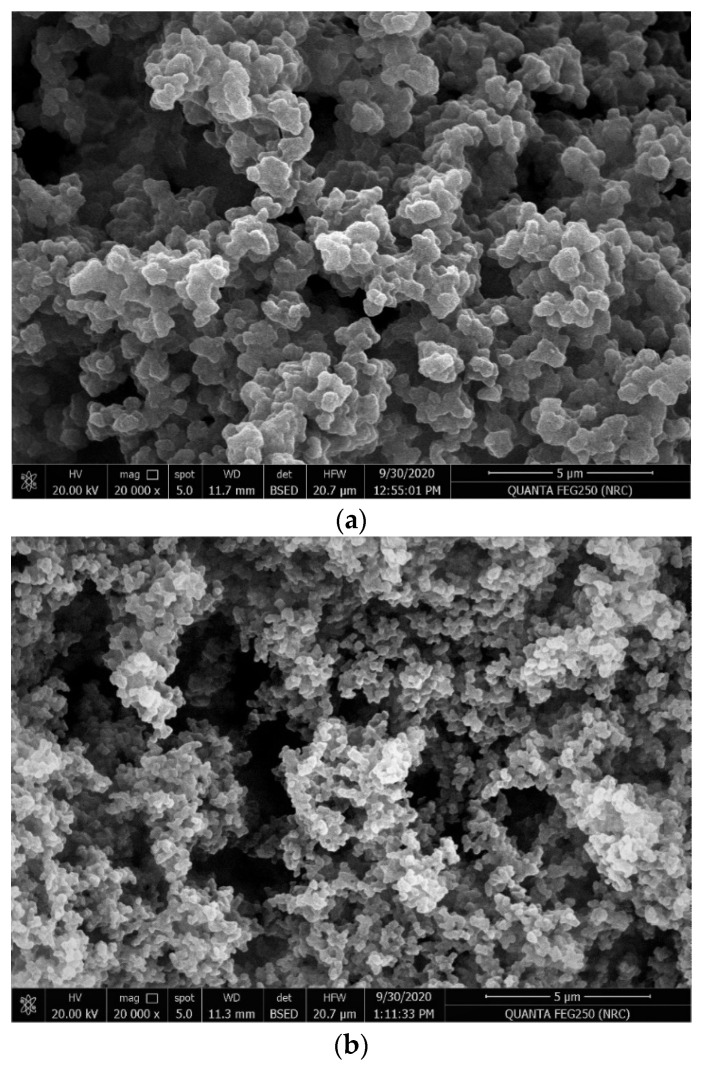
SEM images of (**a**) molecularly imprinted polymer (MIP) and (**b**) nonimprinted polymer (NIP) beads.

Scanning electron microscopy (SEM) was used to investigate the obtained imprinted beads. The SEM image for the MIP beads is presented in Figure 2a. It shows that the MIP beads were uniform with a regular and semispherical shape and had an average diameter of 1 μm. These obtained beads were dispersed well in the polymeric ISE membrane. These particles could lower the membrane resistance and create more binding sites in the membrane [55]. The nonimprinted polymer (NIP) was also prepared in the same way as MIP. It had a morphological structure and particle size distribution similar to those of MIP beads. The SEM image for NIP beads is shown in Figure 2b.

### 3.2. Potentiometric Characteristics of the Sensors

**Figure 3:**
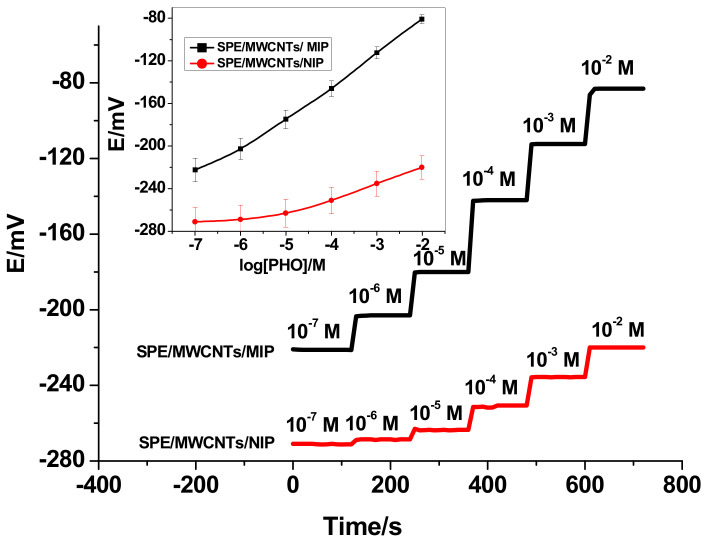
Time-dependent potential responses (inset: the calibration curve for the integrated screen-printed sensors).

The potentiometric response for the all-solid-state screen-printed electrodes was evaluated and tested in solutions with the concentration range of 1.0 × 10^−2^ to 1.0 × 10^−8^ M PHO at pH 5. The dynamic traces for the potential response with the corresponding calibration curve are illustrated in Figure 3. The presented screen-printed electrodes revealed a Nernstian response with a slope of 31.6 ± 0.5 mV/decade (*n* = 5, *R*^2^ = 0.9980) in the range of 5.5 × 10^−6^ to 1.0 × 10^−2^ M with a detection limit 2.5 × 10^−7^ M. To confirm that MIP beads are responsible for the potentiometric response, the response of nonimprinted polymer (NIP)-based sensors (SPE/MWCNTs/NIP) was also investigated. This sensor revealed a sub-Nernstian response with a slope of 14.5 ± 1.2 mV/decade (*n* = 5, *R*^2^ = 0.9950) in the range of 1.0 × 10^−4^–1.0 × 10^−2^ M with a detection limit of 5.0 × 10^−5^ M. This can confirm that the observed response is mainly induced by the specific interaction between PHO molecule and the binding MIP particles in the membrane.

**Figure 4:**
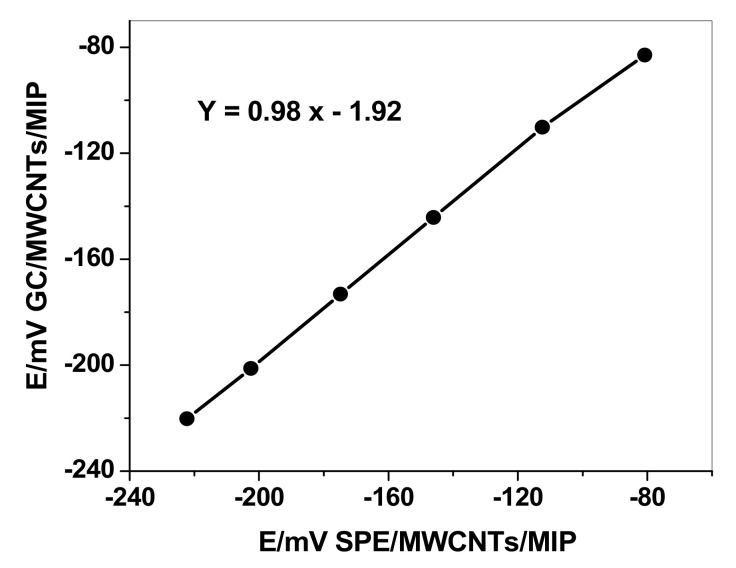
Comparison of potential values obtained for SPE/MWCNTs/MIP and GC/MWCNTs/MIP for different PHO concentrations.

For comparison, PHO ISEs using glassy carbon (GC/MWCNTs/MIP/ISE) as support was fabricated and characterized. The results were compared with those obtained for the integrated screen-printed electrodes (SPE/MWCNTs/MIP/ISE). The GC/MWCNTs/MIP/ISE revealed a sensitivity of 29.9 ± 0.7 mV/decade (*n* = 5, *R*^2^ = 0.997), which was similar to that obtained for the integrated screen-printed electrodes. A plot comparing the potential response values obtained for a calibration curve for PHO concentrations ranging from 1.0 × 10^−7^ M to 1.0 × 10^−2^ M of both GC/MWCNTs/MIP/ISE and SPE/MWCNTs/MIP/ISE simultaneously does not reveal any significant differences (Figure 4). This confirmed that no significant differences were found between screen-printed platforms ISEs and conventional solid-state ISEs in terms of sensitivity and linear range.

The time-response of the proposed sensors was evaluated after measuring the potential corresponding to each corresponding concentration decade (i.e., from 1.0 × 10^−7^ to 1.0 × 10^−2^ M PHO concentration) for 2 min. The time required to attain the equilibrium state was found to be <5 s, which is excellent for the use of these sensors in decentralized analysis. A long-term potential stability test showed a drift of about 0.5 mV/h; this is also satisfactory, considering that the electrode is intended for a single, short reading. 

The potential stability of the presented sensors over different pH values was examined. The sensors showed good potential stability over the pH range of 4 to 6. Below pH 4, an interference from H^+^ causing a positive potential drift was noticed. At pH > 9, the potential response sharply declined due to the formation of the nonionized PHO (i.e., *pKa* = 9.3) [56]. Over the pH range 6.5–8.5, the potential response was false due to the formation of a mixture between mono- and divalent pholcodine [21]. All measurements were carried out in 50 mM MES buffer at pH 5.

Selectivity coefficient values (K*pot*) of the SPE/MWCNTs/MIP/ISE were evaluated using the modified separate solution method [57]. In this method, successive calibration curves were constructed for all interfering ions, starting with the most discriminated ion. The last calibration was carried out with PHO ion. The selectivity coefficient values were then calculated after inserting the extrapolated potentials of each curve at 1 M concentration to the SSM equation:

−log (K*pot*) = (E^o^_1_ − E^o^_2_)/S (1)

where E^o^_1_ and E^o^_2_ are the extrapolated potential readings of the primary and interfering ions, respectively. S represents the slope of the presented sensor (mV/decade). Table 1 displays the values (log *K^Pot^_PHO__,B_* ± standard deviation (*n* = 3)) obtained for the screen-printed PHO sensor.

The selectivity coefficients obtained for the SPE/MWCNTs/MIP indicate that the selectivity of ISEs is related to the sensing membrane itself and obeys the anti-Hofmeister pattern.

### 3.3. Potential Stability

**Figure 5:**
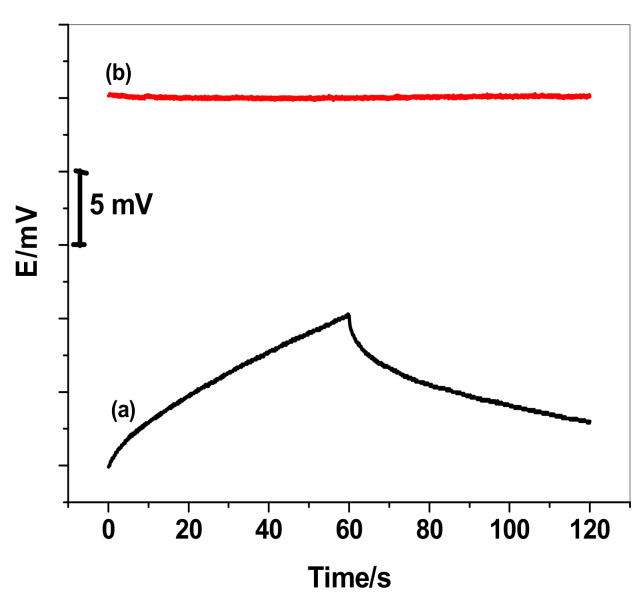
Chronopotentiograms of (**a**) SPE/MIP-ISE and (**b**) SPE/MWCNTs/MIP-ISE.

Short-term potential stability of the SPE/MWCNTs/MIP-ISE was evaluated by carrying out chronopotentiometric measurements in 10 mM PHO solution according to the method presented by Bobacka [58]. Typical chronopotentiograms for SPE/MWCNTs/MIP-ISE and SPE/MIP-ISE are shown in Figure 5. The applied constant current was ±1 nA for 60 s. The potential drift (*ΔE/Δt*) of SPE/MWCNTs/MIP-ISE was found to be 2.3 μV/s (*n* = 3), which is much less than the value (156.2 μV/s) obtained for the SPE/MIP-ISE. Low-frequency capacitances (C) were found to be 423.7 ± 2.3 and 6.4 ± 1.2 μF for SPE/MWCNTs/MIP-ISE and SPE/MIP-ISE, respectively.

The adhesion between the ion-sensing membrane and conducting substrate for a long time can cause a potential response deterioration [59]. Therefore, the long-term potential stability and lifetime of the presented electrodes were investigated. The test was carried out by continuously measuring the potential corresponding to 10 mM PHO solution for 24 h. The potential drift was found to be 18.3 ± 2.5 μV/h (*n* = 3), indicating excellent long-term stability of the SPE/MWCNTs/MIP-ISE. Both short-term potential stability and long-term potential stability were greatly enhanced by the insertion of the MWCNT layer between the sensing membrane and electronic conductor substrate. This enhanced potential stability is mainly due to the high double-layer capacitance of the MWCNTs.

The electrode lifetime was evaluated by measuring the potential response of the same electrode when inserted in 10 mM PHO solution for 15 days (*n* = 3). The limit of detection and the slope-sensitivity of the SPE/MWCNTs/MIP-ISE changed after continuous measurements for 2 weeks to be 5 × 10^−4^ M and 16.2 ± 0.9 mV/decade, respectively.

### 3.4. Effects of Light, O_2_, and CO_2_

**Figure 6:**
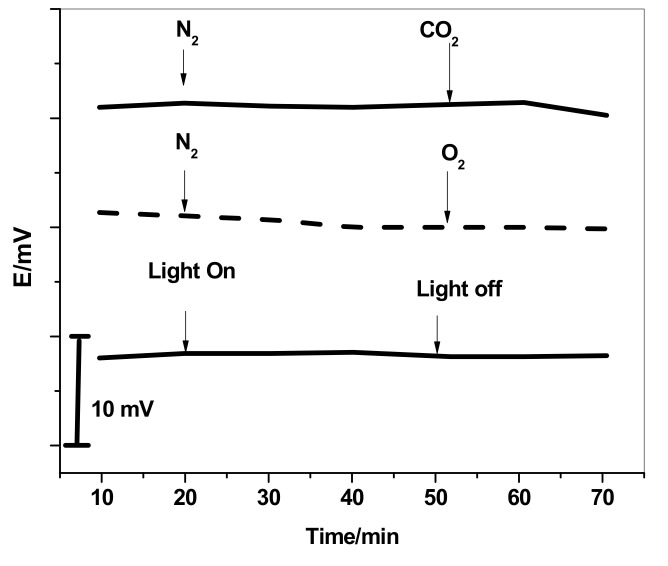
Robustness of the SPE/MWCNTs/MIP-ISE.

To test the robustness of the SPE/MWCNTs/MIP-ISE, the effects of different gases, namely CO_2_, O_2_, and N_2_, and light on the potential stability were studied. The test was carried out by measuring the potential response of the sensor in 10 mM PHO solution and bubbling the above-mentioned gases for 30 min. The influence of light was evaluated after immersing the MWCNT-based electrode in 10 mM PHO solution with the ambient light on/off. As can be seen in Figure 6, no potential drifts were observed during the presence of such effects. This indicates that the presented electrode is robustly resistant to interference from CO_2_, O_2_, N_2_, and light. 

### 3.5. Water-Layer Test

**Figure 7:**
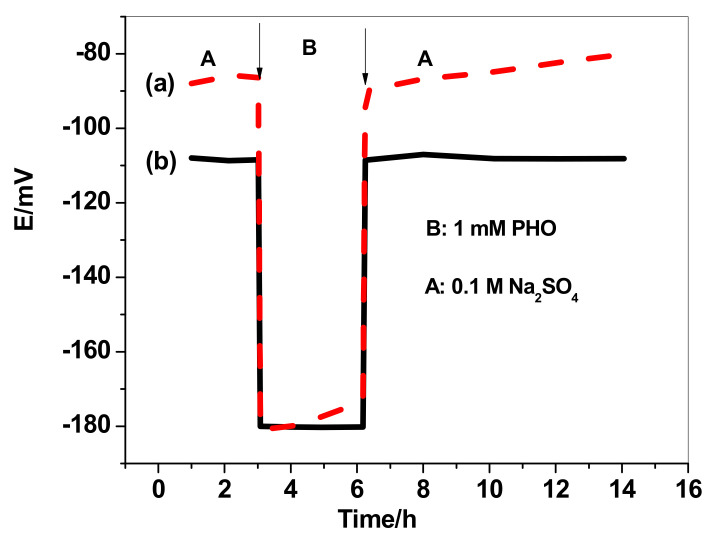
Water-layer tests for both (**a**) SPE/MIP-ISE (dotted line) and (**b**) SPE/MWCNT/MIP-ISE (solid line).

The nonexistence of water-layer formation between the sensing membrane and the conducting substrate in presence of the MWCNT layer was also investigated. Both SPE/MWCNTs/MIP-ISE and SPE/MIP-ISE were sequentially immersed in 1 mM PHO, 0.1 M NaCl, and 10 mM PHO. As shown in Figure 7, SPE/MWCNTs/MIP-ISE revealed a stable potential response as compared with the SPE/MIP-ISE. This demonstrates the nonexistence of a water layer at the interface between the sensing membrane and either MWCNTs or the electronic conductor substrate. This is attributed to the high hydrophobicity of MWCNTs.

### 3.6. Analytical Applications

To test the applicability of the presented sensors, different serum samples were spiked with known amounts of PHO, and the pH was adjusted to pH 5 using 50 mM MES buffer solution. Five samples containing PHL concentration in the therapeutic range were analyzed using the presented potentiometric procedure, and the results were then compared to those obtained with the standard chromatographic method suggested by the British Pharmacopeia [60]. All results are presented in Table 2. No significant difference between the values at 95% confidence was noticed.

For further applications, the suggested potentiometric method was used for the determination of PHO in different pharmaceutical formulations containing pholcodine. The samples include syrups (Cyrinol, Apic Pharm. Co., Cairo, Egypt; labeled 4 mg PHL/mL) and suspensions (Marynol, Glaxo Wellcome, Cairo, Egypt; labeled 4 mg/mL). The samples were analyzed by the proposed potentiometric method and the reference method suggested by the British Pharmacopoeia. All results are shown in Table 3. The data obtained confirmed the validity of using the presented sensors for the routine assessment of PHO.

## 4. Conclusions

This work presents the successful development of an all-solid-state ISE for pholcodine determination. The proposed sensors are extremely simple to design and cost-effective, and they have a fast response. The device was successfully used for pholcodine determination in spiked serum samples and in different pharmaceutical dosages containing pholcodine. The results obtained were compared with those obtained with more sophisticated lab-based approaches, such as high-performance liquid chromatography (HPLC). Among the most attractive features, the presented potentiometric method works with very small sample volume without further sample pretreatment. The method can be considered as an ideal addition to the growing field of analytical platforms based on screen printing.

## Figures and Tables

**Table 1:** The selectivity coefficients (log K*^Pot^_PHO,B_*) of SPE/MWCNTs/MIP.

Interfering Ion, B	log K*^Pot^_PHO,b_ ±* SD *
Morphine	−1.5 ± 0.2
Ethylmorphine	−1.7 ± 0.4
Ephedrine	−3.5 ± 0.3
Codeine	−3.7 ± 0.2
Dextromethorphan	−4.1 ± 0.5
Carbinoxamine	−4.5 ± 0.3
Caffeine	−4.7 ± 0.4
Ketamine	−4.5 ± 0.6
K^+^	−5.1 ± 0.4
Ca^2+^	−5.5 ± 0.2
Na^+^	−5.2 ± 0.1

* Standard deviation of three measurements.

**Table 2:** Determination of PHO in spiked serum samples.

Sample	Amount Spiked, µM	Amount Found, µM ± SD *				F-Test
		Potentiometry	Recovery, %	Reference Method, [60]	Recovery, %	
1	1.0	0.94 ± 0.04	94.0	0.97 ± 0.01	97	2.21
2	2.0	1.91 ± 0.3	95.5	1.98 ± 0.2	99.0	3.55
3	3.0	2.73 ± 0.4	91.0	3.02 ± 0.3	100.6	4.25
4	5.0	4.65 ± 0.5	93.0	4.92 ± 0.1	98.4	2.35
5	10.0	9.54 ± 0.4	95.4	10.13 ± 0.3	101.3	4.11

* Standard deviation for average of 5 measurements.

**Table 3:** Determination of PHL in different pharmaceutical formulation samples.

Sample	Labeled Amount, mg/mL	Amount Found, mg/mL ± SD *				F-Test
		Potentiometry	Recovery, %	Reference Method, [60]	Recovery, %	
Cyrinol, Apic Pharm. Co., Egypt (Syrup)	4.0	3.62 ± 0.2	90.5	3.98 ± 0.2	99.5	1.61
Marynol, Glaxo Wellcome, Egypt (Suspension)	4.0	3.74 ± 0.3	93.5	3.93 ± 0.3	98.2	3.24

* Standard deviation for average of 5 measurements.

## Data Availability

Not applicable.
